# Comparative Analysis of Flower Volatiles from Nine Citrus at Three Blooming Stages

**DOI:** 10.3390/ijms141122346

**Published:** 2013-11-13

**Authors:** Muhammad Azam, Min Song, Fangjuan Fan, Bo Zhang, Yaying Xu, Changjie Xu, Kunsong Chen

**Affiliations:** Laboratory of Fruit Quality Biology/The State Agriculture Ministry Laboratory of Horticultural Plant Growth, Development and Quality Improvement, Zhejiang University, Zijingang Campus, Hangzhou 310058, China; E-Mails: azam@zju.edu.cn (M.A.); 21116061@zju.edu.cn (M.S.); 21116086@zju.edu.cn (F.F.); bozhang@zju.edu.cn (B.Z.); xuyaying@zju.edu.cn (Y.X.); akun@zju.edu.cn (K.C.)

**Keywords:** citrus types, volatiles, unopened flower, half opened flower, fully opened flower, HS-SPME, GC-MS

## Abstract

Volatiles from flowers at three blooming stages of nine citrus cultivars were analyzed by headspace-solid phase microextraction (HS-SPME)-GC-MS. Up to 110 volatiles were detected, with 42 tentatively identified from citrus flowers for the first time. Highest amounts of volatiles were present in fully opened flowers of most citrus, except for pomelos. All cultivars were characterized by a high percentage of either oxygenated monoterpenes or monoterpene hydrocarbons, and the presence of a high percentage of nitrogen containing compounds was also observed. Flower volatiles varied qualitatively and quantitatively among citrus types during blooming. Limonene was the most abundant flower volatile only in citrons; α-citral and β-citral ranked 2nd and 3rd only for Bergamot, and unopened flowers of Ponkan had a higher amount of linalool and β-pinene while much lower amount of γ-terpinene and *p*-cymene than Satsuma. Taking the average of all cultivars, linalool and limonene were the top two volatiles for all blooming stages; β-pinene ranked 3rd in unopened flowers, while indole ranked 3rd for half opened and fully opened flower volatiles. As flowers bloomed, methyl anthranilate increased while 2-hexenal and *p*-cymene decreased. In some cases, a volatile could be high in both unopened and fully opened flowers but low in half opened ones. Through multivariate analysis, the nine citrus cultivars were clustered into three groups, consistent with the three true citrus types. Furthermore, an influence of blooming stages on clustering was observed, especially with hybrids Satsuma and Huyou. Altogether, it was suggested that flower volatiles can be suitable markers for revealing the genetic relationships between citrus cultivars but the same blooming stage needs to be strictly controlled.

## 1. Introduction

Floral fragrance has been studied over the last decade to understand the biosynthesis, emission, regulation and ecological impacts of emitted floral volatiles [1]. Flower volatiles play important roles particularly in communication between flowering plants and their pollinating agents [2,3], and repelling of herbivores [4,5]. Flower volatiles are also important due to their commercial application in food, pharmaceutical, perfume, and cosmetic industries [6,7].

Plants produce volatiles from various biosynthetic pathways such as terpenoids, benzenoids, aliphatics, aromatics and fatty acid derivatives [8,9]. Terpenoids are the largest class of plant secondary metabolites, having many volatile representatives, and are important components of floral scent in a wide range of species [10]. They represent the largest and most diverse family of natural products, including over 30,000 individual compounds, half of which can be synthesized by plants [11]. Terpenoids are produced from basic C_5_ units isopentenyl diphosphate (IPP) and dimethylallyl diphosphate (DMAPP), through serial formation of geranyl diphosphate (GPP), farnesyl diphosphate (FPP), and geranyl geranyl diphosphate (GGPP) via elongation reactions [12,13], to generate the precursors of monoterpenes, sesquiterpenes, and diterpenes. The conversions of these precursors are carried out by a large family of enzymes known as terpene synthases (TPS), to produce a vast range of terpenoids [14,15].

Germplasm diversity affects agricultural development, sustainability and food security [16], and germplasm studies can provide a great deal of knowledge about the origin, development of plant material and collection of plant genetic resources. For citrus, the importance for collection, preservation, and evaluation of genetic resources for industry has been well described [17]. Citrus belongs to a large family the Rutaceae, and a diverse range of taxonomic classification systems has been proposed for the subgenus citrus [18,19], with varying numbers of citrus species. There is a large amount of variation among citrus types and cultivars because of frequent bud mutation, interspecific and intergeneric hybridization, apomixis, polyembryo and a long history of cultivation [20].

Chemical composition has also been employed in devising taxonomic classification. Studies have previously been conducted on leaf and peel essential oil for the classification of citrus cultivars [16,21,22], fruit juice [23] and blossom volatiles [24]. Chemotaxonomic studies have been useful in distinguishing the phytoplankton communities in aquatic systems, which is an economical approach that bears high advantageous for study of a large ecosystem [25]. Li *et al.* investigated the evolution and classification of bamboos, and the results derived from leaf wax n-alkanes were consistent with those from morphological characters [26]. Recently, Liu *et al.* studied the classification of citrus and its related genera based on chemotaxonomic analysis from citrus leaf and peel oils, and the results were consistent with morphological and genetic characteristics [16]. These studies suggested that chemotaxonomic analysis was a reliable, revealing a powerful research tool for taxonomic studies.

As far as we know, neither the evolution of chemical composition of volatiles nor their chemotaxonomic studies have been reported for flower development from various citrus types in previous studies. In general, studies concerning the citrus flower volatiles are rather scarce and substantial differences could be outlined due to genetic material, environmental conditions, analyzed stage/organ as well as sample preparation and analytic methods used for studies [27].

With these considerations in mind, the objectives of this study were to investigate the volatile profiles of flowers during blooming stages of nine citrus cultivars from five genetic types. Differences were observed both among citrus cultivars and during blooming stages. This study may reveal new clues to the evolution of flower volatile during blooming and comprehensive information of detected flower volatiles for further use, such as citrus breeding programs or in the cosmetic industry.

## 2. Result and Discussion

### 2.1. Analysis of Citrus Flower Volatiles by HS-SPME-GC-MS

In this study, a total number of 110 flower volatiles were detected by headspace solid phase microextraction and gas chromatography spectrometry (HS-SPME-GC-MS) from different citrus types at three blooming stages (Table 1). HS-SPME is a simple and efficient method with solventless extraction technique as compared to conventional extraction methods that involved complex sample preparation and large amounts of solvent [28–30]. Moreover, this technique is sensitive enough to identify plant odors from specific tissues [31], such as flower organs, pollen [32], and nectar [32]. The fiber with three components divinylbenzene/carboxen/polydimethylsiloxane (DVB/CAR/PDMS) has been extensively used due to its high ability to extract a large number of volatiles compared to other fibers [21–33], and therefore was employed in the current study.

The percentage and retention indices of identified compounds are listed in Table 2, where the reported volatiles and their amounts are presented as peak area percentage for each cultivar and each stage. Compounds detected belonged to different chemical classes; aldehydes (9), monoterpene hydrocarbons (17), oxygenated monoterpenes (26), sesquiterpene hydrocarbons (32), oxygenated sesquiterpenes (6), ketones (2), esters (4), and miscellaneous (14) (Table S1). The number of volatiles differed according to the stage of flower blooming and cultivar. Of 110 volatiles detected, 68 were identified and reported in previous studies [32,34–39], while the other 42 have not been reported previously (Table S1). This might be explained by the more advanced analysis techniques or wide range of citrus cultivars or larger number of blooming stages used in this study.

### 2.2. Variation in Total Flower Volatile Amount

**Figure 1:**
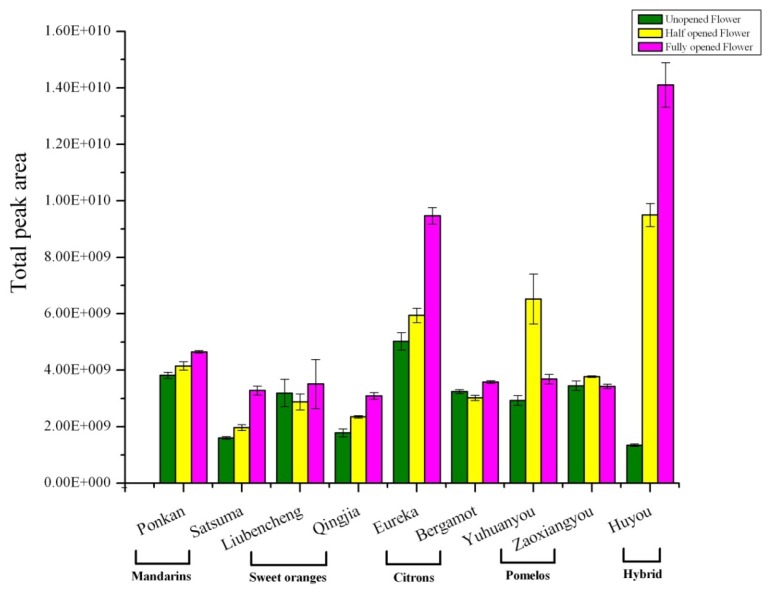
Total volatile contents of unopened, half opened and fully opened flower of five citrus types.

The changes in total volatile amount in different citrus flowers during blooming are shown in Figure 1. For flowers at the fully opened stage, Huyou produced the highest amounts, followed by lemon, while the rest were similar to each other. On the whole, fully opened flowers had a higher volatile amount than other stages, this is especially obvious for Huyou and lemon (Figure 1). This observation could indicate that during full blooming, the increased emission of volatiles might be helpful for attracting pollination agents. Flower volatiles have been infrequently studied in citrus previously, Jabalpurwala *et al.* reported that pomelo produced the highest total level of volatiles followed by grapefruit, sweet orange, mandarin and the lowest in lime [24]. With different cultivars in this study, we observed that Huyou produced an even higher amount of flower volatiles than the two pomelos tested. Huyou is a natural citrus hybrid, with parents tentatively identified as pomelo and sour orange [40], originating in Changshan county of Zhejiang province. Furthermore, the total ion profile from GC-MS and volatile fingerprints obtained from electronic nose emitted from different cultivars during development also changed quite significantly flower stages and citrus types (Figure S1).

### 2.3. Changes in Chemical Classes of Volatiles Produced during Flower Development

**Figure 2:**
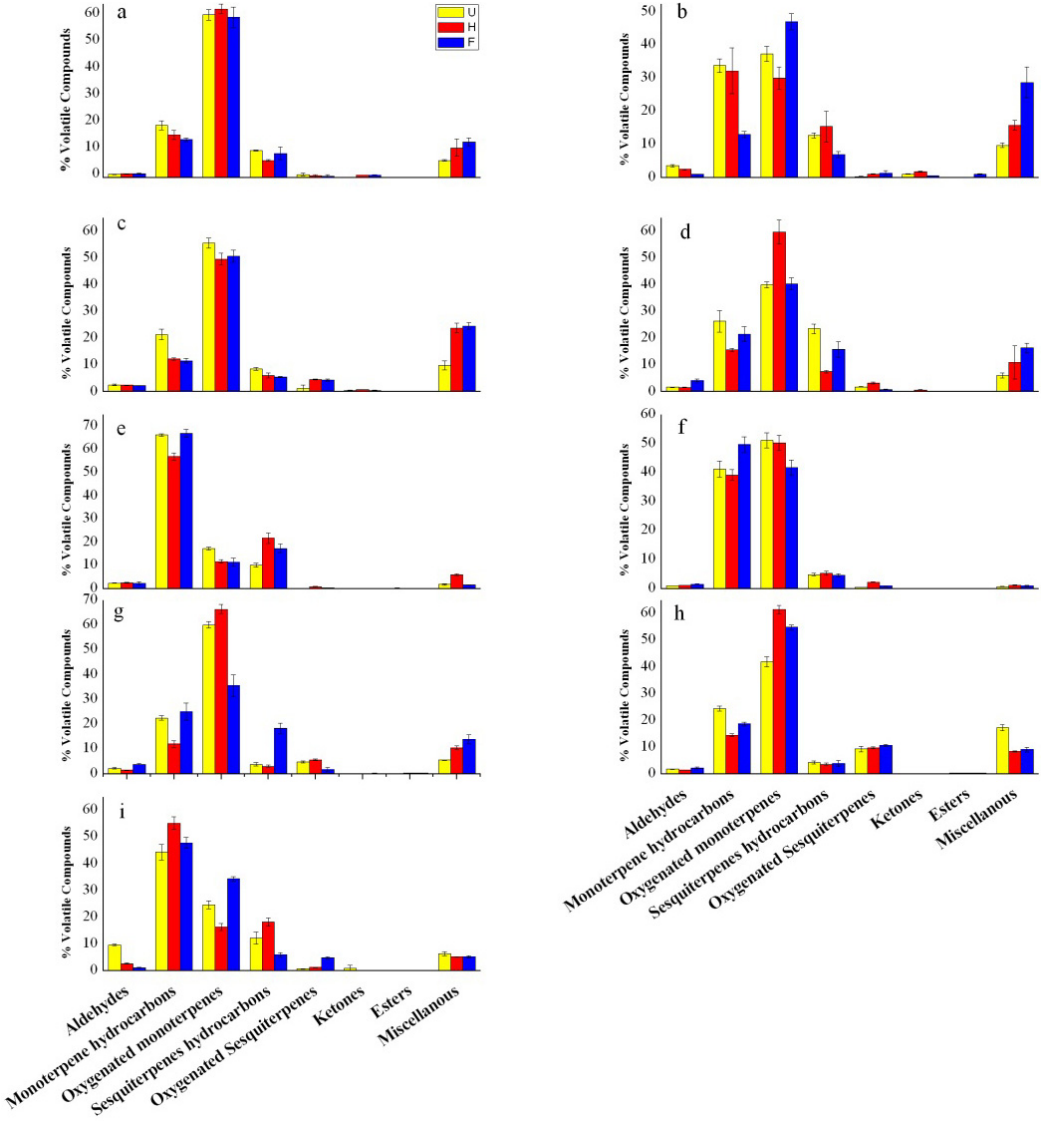
Evolution of each group of flower volatile compounds during blooming in the Ponkan (**a**); Satsuma (**b**); Qingjia (**c**); Liubencheng (**d**); Eureka (**e**); Bergamot (**f**); Yuhuanyou (**g**); Zaoxiangyou (**h**); and Huyou (**i**); Citrus flowers were sampled at unopened flower “U”, half opened “H”, and fully opened flower “F” blooming stages.

HS-SPME-GC-MS analysis of flower volatiles indicated that the percentage of each chemical class making up the total volatiles varied among genetic types and cultivars. It was found however, that the differences in the major classes of volatiles were not significantly changed during blooming in most cultivars (Figure 2).

Monoterpenoids were the major volatile classes in flowers, regardless of stage and cultivar, which accounted for at least 60.20% of total volatiles, and was as high as 92.78% in Bergamot due to the high percentage of monoterpene hydrocarbons and oxygenated monoterpenes (Figure 2), which was known by the name “finger citron”, a citrus with flower and fruit for ornamental as well as medicinal purposes. In addition, sesquiterpenoids were presented in large numbers but in low amounts in almost all cultivars, while few ketones and esters were evident in citrus flower volatiles. The trend in evolution of major chemical classes was not consistent among all cultivars, and fluctuated due to changes in content of major compounds. A similar observation has been reported for the volatile constituents of *Rosa canina* L. during flower development [41] and also from leaf volatiles of different citrus during leaf development [21].

### 2.4. Variation in Volatile Compounds from Nine Citrus Cultivars

Flower volatiles showed wide variation in different citrus types and during blooming (Table 2). In order to rank the major volatiles, the average percentage of individual volatiles was calculated among all cultivars at each stage. The top 15 volatiles, on average of all nine citrus, are shown in Tables 3–5. It was found that linalool and limonene were the top two for all blooming stages, while β-pinene ranked third in unopened flowers, whereas indole ranked third for half opened and fully opened flower volatiles (Tables 4 and 5). It is interesting to note the existence of obvious differences in accumulation of major flower volatiles between cultivars among five citrus types. For example, limonene was the most abundant flower volatile in Eureka and Bergamot for all blooming stages but was not in the top two for other citrus; α-citral and β-citral ranked second and third for all blooming stages in Bergamot, while these two were generally low in other citrus, except for half opened flower of Qingjia; indole ranked in the top nine in all citrus except for two citrons (Tables 3–5). Differences also existed between cultivars of the same type. For example, linalool and β-pinene, by percentage, were about one and two fold higher in unopened flowers of Ponkan than Satsuma, whereas, γ-terpinene and *p*-cymene was about six and fourteen fold lower (Table 3). Methyl anthranilate was abundant in half opened and fully opened flowers of Satsuma but was over 20 times less in flowers of Ponkan at the same blooming stage (Tables 4 and 5). In orange, β-pinene was abundant in Liubencheng but was absent from flower volatiles of Qingjia for all blooming stages (Tables 3–5). In half opened and fully opened flowers of pomelos, methyl anthranilate was abundant in Yuhuanyou but absent in Zaoxiangyou, while the situation was reversed for (*E*)-ocimene (Tables 4 and 5).

When the profiling of flower volatiles was compared with leaf volatiles of the same citrus types from our previous studies [21], some similarities were observed. Seven volatiles, linalool, limonene, β-pinene, γ-terpinene, β-elemene, α-citral and β-citral, were present as the top ten volatile in leaves and flowers at all stages, especially, linalool and limonene, in the top five in all samples analyzed (Tables 3–5) [21]. On the other hand, some striking differences were also observed between leaf and flower volatiles. For example, (*E*)-ocimene ranked second, on average for all citrus examined, both in young and mature leaves but ranked from 4th to 13th in flowers; indole ranked in the top nine in all citrus except for two citrons, in flower volatiles, while it was only identified in traces from leaf of Satsuma and Hongshigan; β-terpinene ranked in the top six in leaves but was not detected in flowers; Methyl anthranilate was abundant in volatiles of half or fully opened flowers of Satsuma, Liubencheng, Yuhuanyou and Huyou but was not detected in leaves (Tables 3–5) [21].

Here in this study, linalool and limonene were the major flower volatiles in most cultivars, which may play important roles in attraction of pollinators and as defense compounds against herbivores and pathogens [21,42,43]. As to the occurrence of numerous volatile components belonging to different chemical classes, it has been reported that this is related to the operation of different metabolic pathways [41,44]. The increase or decrease in amount of volatiles during flower blooming stages might suggest an activation or synthesis of related terpene synthases which catalyze formation of the critical intermediate α-terpinyl or pinyl cation for cyclic monoterpene and geranyl or linalyl cation for acyclic monoterpenes [15].

### 2.5. Changes in Volatile Abundance during Flower Blooming in Nine Cultivars

**Figure 3:**
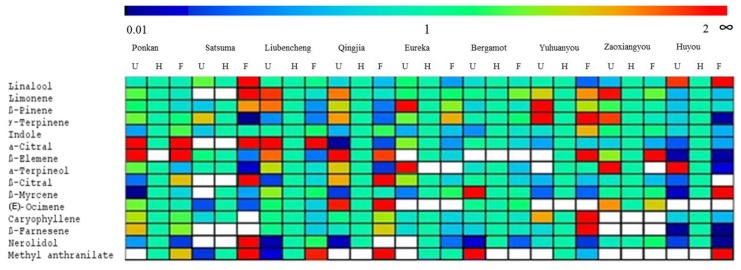
Ratio of major flower volatiles in either unopened (U) or fully opened (F) to half opened (H) flowers from nine citrus cultivars. Color code shown above the figure: red shows high, blue low. The blank cells indicate volatiles that were absent.

Flower volatile profiles varied during blooming, and the changes in major volatiles are indicated in Tables 3–5. The ratios of the content, by percentage, in unopened and fully opened flowers were calculated with respect to half opened flowers (Figure 3). For some volatiles, similar trends were observed in various cultivars, especially for those from the same genetic type. For example, average percentages of methyl anthranilate were not ranked in the top 15 volatiles in unopened flowers, but ranked 9th in half opened ones and 4th in fully opened ones, and this was also generally true for individual cultivars except for Eureka and Yuhuanyou; 2-hexenal and *p*-cymene existed as a major volatile in unopened flowers but was not a major one, on average, in flowers of the other two stages studied (Figure 3, Tables 3–5). However, it was also revealed that the ratio changes were inconsistent among cultivars. For example, linalool decreased in some citrus, especially in Yuhuanyou and Eureka, during flower blooming, while it increased in some other citrus, such as Satsuma and Huyou. Similarly, limonene and β-pinene exhibited higher amounts in unopened flowers of some cultivars but in fully opened flowers of some other cultivars. Interestingly, the amount of some volatiles, such as linalool in Huyou, γ-terpinene in Yuhuanyou, α-citral in Ponkan and Liubencheng, as well as β-elemene and (*E*)-ocimene in Qingjia, was high in both unopened and fully opened flowers but was low in half opened ones (Figure 3). These results provide clear evidence that the content of some major volatiles did not consistently increase during flower blooming, and that volatiles have a great influence during flower maturity, probably related to their different roles during development such as protection against pathogen and attraction of pollinating agent [27].

### 2.6. Multivariate Analysis of Flower Volatiles during Development from Nine Cultivars

**Figure 4:**
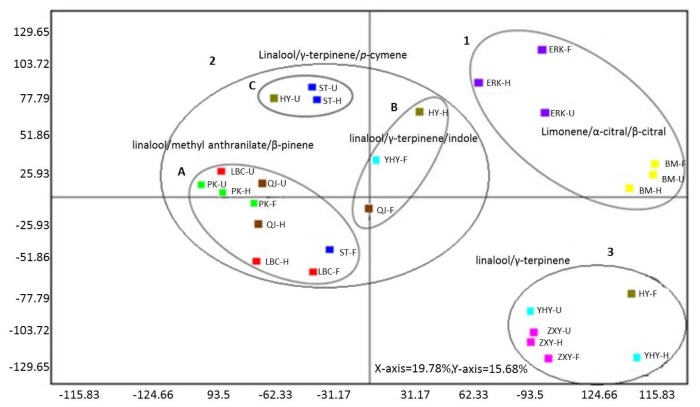
Principle component analysis (PCA) of flower volatiles from nine citrus cultivars during blooming (abbreviated names of cultivars are presented in Table 1; each color represents one cultivar; while U, H and F represents unopened, half opened and fully opened flower stages). Note: each subgroup exhibited abundant volatiles.

**Figure 5:**
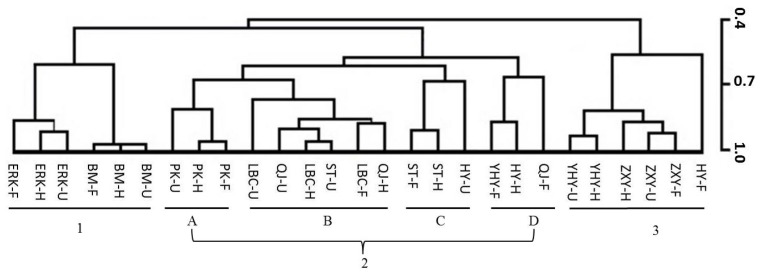
Hierarchical cluster analysis based on flower volatiles during blooming for nine cultivars (abbreviated names of cultivars are as in Table 1, and **1** to **3** represent different cluster groups; while U represents unopened; H: half opened and F: fully opened flower blooming stages). Group **1** represented the citron, group **2** comprised four subgroups (2**A** Ponkan, 2**B** sweet orange and Satsuma mandarin, and 2**C** Satsuma mandarin and Huyou, and 2**D** Yuhuanyou, Huyou and Qingjia), while group **3** represented pomelo and Huyou.

To evaluate the genetic and developmental influences on flower volatiles, principle component analysis (PCA) and hierarchical cluster analysis (HCA) were performed. The PCA horizontal axis explained 19.78% of total variance and the vertical axis a further 15.68%, and three major groups, with the mandarin (2A, 2B, 2C), citron (1) and Pomelo (3) clearly separated, were observed (Figure 4), suggesting the existence of three major clusters with their subgroups as mentioned previously, and each subgroup was dominated by presence of specific major volatiles (Figure 4). More details were revealed by the HCA data, which also indicated that the nine cultivars could be clustered into three groups (Figure 5).

Group 1 contains all citrons, all the samples yielded volatiles which were clearly differentiated from the other cultivars due to high amounts of limonene, which was the most abundant flower volatile in citrons (Tables 3–5). Group 2, comprising four subgroups, represented the volatiles profiling of Ponkan (2A), sweet oranges and Satsuma (F) (2B), Satsuma and Huyou (2C), as well as containing the volatiles of Yuhuanyou, Huyou and Qingjia (2D). Group 3 contains only pomelos and Huyou. These results are in agreement with the previous studies that the cultivars can be classified based on morphological and biochemical characteristics with citron, mandarin and pomelo identified as the only true citrus types [20,24,45]. The origin of other citrus, as hybrids between true citrus types, has been traced traditionally by molecular markers [20], and in this study, it was observed that the origin can be explored by volatile profiles as well. For example, Satsuma mandarin was clustered with sweet orange in subgroup 2B, rather than with Ponkan, which was in subgroup 2A. This supported the viewpoints of previous studies [46–48] where it has been reported that Satsuma mandarin is a hybrid and more closely related to sweet orange or pomelo rather than to mandarins, which is also consistent with the Tanaka classification [45], which classified Satsuma mandarin as separated from Ponkan. Flower volatile profiling data was also helpful for parentage analysis. For example, Huyou, which has been proposed to be a hybrid between a pomelo and a sour orange based on results from internal transcribed spacer (ITS) sequencing [40], was observed to be related to pomelos and a citrus belong to subgroup 2C, and the possibility that a sour orange could be clustered into this subgroup is worthy of further examination. Furthermore, it can also be concluded that volatile profiles of a hybrid could show different degree of contribution from its parents at different blooming stages. For example, the profiles of flower volatiles of Huyou at unopened, half opened, and fully opened stages were similar to those of a citrus in subgroup 2C, 2D and group 3, respectively. Similar clustering was also observed in Satsuma. Altogether, all these results confirmed that citron, pomelo and mandarins were the three basic species of cultivated citrus, and indicated that the genetic influences on HCA analysis could be stronger than geographical and temporal factors, which was in accordance with the previous studies [16,19–21,24]. In our other recent study, leaf volatiles of nine citrus were analyzed and similar conclusions and implications were produced, although to a lesser extent [21].

However, the influence of blooming stages on cluster analysis varied greatly among different citrus. For true citrus types, citrons, true mandarin (Ponkan), and to a lesser extent, pomelos, volatiles from all blooming stages clustered together (Figure 5), while for Satsuma, flower volatiles at unopened and half opened stages were clustered together in a same subgroup (2C), but at full opened stage clustered with another subgroup (2B) (Figure 5). This might be due to the almost doubled amount of linalool, 15-fold increment in methyl anthranilate, the second most abundant volatile in full opened flower, as well as a sharp decrease in γ-terpinene, dropping from the second rank down to the 34th, and *p*-cymene, falling from the third rank to undetectable during flower blooming (Tables 3–5). Similarly, unopened Huyou flowers emitted volatiles close to mandarins/sweet oranges, while half or fully opened flowers had volatiles closer to pomelos (Figure 5), which could have resulted from the substantial increase in linalool and methyl anthranilate as well as sharp decrease in γ-terpinene and *p*-cymene (Tables 3–5). All these data indicated that for some citrus, especially those hybrids, the flower volatiles profiles could vary greatly during blooming, and equivalent blooming stages need to be strictly compared when using flower volatiles as a marker for revealing the genetic relationships between citrus cultivars. Genetic diversity has been recently estimated based on volatile compounds from both fruit peel and leaf of citrus and its relatives suitable for interspecies phylogenetic studies [16], based on EST-SSR markers [49], and also chemical polymorphism of citrus leaf volatiles of different citrus types [21]. Our results, in relation to previous studies, present new insights into flower volatiles variability among citrus types, and could be helpful for characterization of citrus cultivars based on flower volatiles during blooming.

## 3. Experimental Section

### 3.1. Materials

Flower samples of nine citrus cultivars from five citrus types (Table 1) were collected in 2011 from research orchards of adult (10–15 years old) healthy trees, uniform in growth, size and vigor, at Wenzhou, Huangyan, and Wenling cities of Zhejiang Province, China. Flowers were collected during full blossom and separated into three distinct stages, *i.e.*, unopened flower, half opened and fully opened. The samples were stored in dry ice after collection and transferred to the laboratory in 4 h, and immediately immersed into liquid nitrogen and kept at −80 °C until analysis. Three biological replicated were collected for each cultivars from nine plants, using three plants as biological replicates.

### 3.2. Headspace Extraction of Flower Volatiles

Samples were ground in liquid nitrogen, one gram powder was weighed and put into a 10 mL glass vial. Before capping, five milliliter of saturated sodium chloride solution, for stopping enzymatic degradation and helping to drive the volatiles into headspace, fifty microliter of internal standard solution (1-hexanol, 0.1%, *v*/*v*), as well as a magnetic stirrer bar were added to the vial. The vial was heated at 40 °C for 30 min on a heating platform with continuous agitation at 600 rpm. The SPME, 50/30 μm CAR/DVB/PMDS fibers (Supleco, Bellefonte, PA, USA) were preconditioned according to the manufacturer’s instructions, then inserted into the headspace, and extraction was continued for 30 min under the same conditions (40 °C, 600 rpm). The fiber was subsequently desorbed in an injector for 5 min.

### 3.3. Gas Chromatography with Mass Spectrometry (GC-MS) Analysis

Citrus samples were subjected to analysis by GC-MS (7890A GC, 5957C inert XL MSD with triple-axis detector, Agilent Technologies, Santa Clara, CA, USA) with HP-5MS capillary column (5% Phenyl methyl siloxane, 30 m × 0.25 mm i.d., 0.25 μm film thickness; J & W Scientific, Folsom, CA, USA) for separation and analysis of headspace volatiles. The carrier gas was helium at a flow rate of 1 mL/min. Samples were injected by desorbing the SPME fiber at the GC injection port at 250 °C with splitless mode. The oven starting temperature was 40 °C, which was held for 3 min, then raised to 130 °C at a rate of 3 °C/min and held for 13 min, again ramped up to 230 °C at a rate of 15 °C/min and finally held for 8 min. The data from the mass spectrometer in the electron impact mode (MS/EI) at 70 eV was recorded in the range *m*/*z* 35 to 350. The mass spectrophotometer was operated in the selective ion mode under autotune conditions and the raw data obtained from GC-MS were processed with AMDIS and Enhanced Chemstation software for GC-MS (Agilent G1701EA MSD).

### 3.4. Identification of Volatiles

The volatiles from citrus were identified on the basis of their mass spectra obtained from GC-MS, retention indices, retention time and data library of GC-MS. The retention indices were determined in relation to homologous series of *n*-alkanes (C7–C40) (SUPELCO-USA) under the same operating conditions. Identification of volatiles was preliminarily based on retention indices (RI) from the literature, retention time (RT) with those of authentic standards available, and tentative identification was achieved by matching the mass spectra and RI. Further identification was based by matching mass spectral fragmentation patterns with those stored in NIST/EPA/NIH Mass Spectral Library (NIST-08) of GC-MS data systems. Relative percentage amounts of the identified compounds were obtained by normalizing the data using the internal standard methods.

### 3.5. Electronic Nose Measurements

Flower fingerprints were evaluated by electronic nose (FOX 4000, Alpha MOS, Toulouse, France) equipped with 18 metallic oxide sensors according to the method of Zhang *et al.* [50]. Briefly, one gram of flowers was ground to a fine powder in liquid nitrogen, 5 mL saturated sodium chloride solution, used to drive the volatiles into headspace, and mixed in a 10 mL tube. For electronic nose measurements, 2 mL of the prepared homogenate were then transferred and sealed in a 10 mL vial, heated at 40 °C for 30 min, and finally 2 mL of headspace gas was injected for analysis. The signal acquisition lasted for 2 min, and was followed by 4 min for baseline recovery. A diagnostic test was conducted according to the manufacture’s recommendations to check the performance of metallic oxide sensors and to avoid base line drift.

### 3.6. Statistical Analysis

Citrus flower volatiles were obtained from the total ion current chromatogram (TIC) generated by GC-MS. The peak areas of all the compounds relative to internal standard (1-hexanol) were used to calculate the percentage of individual volatiles. Data were transformed via log 2 with MultiExperiment Viewer (MeV_4.8.1) (http://www.tm4.org, Dana-Farber Cancer Institute, Harvard Medical School, Boston, MA, USA) for analyzing the chemical variability of flower volatiles among different samples. For hierarchical cluster analysis (HCA) and principle component analysis (PCA), mean values of volatiles were employed in each sample. The average linkage clustering was performed based on Pearson correlation (16). This method is very useful to identify trends of chemical variables from different samples.

## 4. Conclusions

The profiling of flower volatiles from nine citrus cultivars at three blooming stages revealed significant differences between cultivars and blooming stages. Monoterpenoids, linalool and limonene, were the major flower volatiles, followed by indole, β-pinene, α-citral, and γ-terpinene. Flower volatiles from each cultivar exhibited a characteristic profile of volatiles that contribute to its unique aroma attributes. Citrons were rich in limonene and Bergamot was rich in α-citral and β-citral. As flowers bloom, volatiles like methyl anthranilate increased while some others like 2-hexenal and *p*-cymene decreased, and some volatiles in a specific citrus, like linalool in Huyou as well as γ-terpinene in Yuhuanyou, could be high in both unopened and fully opened flowers but low in half opened ones. Multivariate analysis data were consistent with the theory of three true citrus types, and supported the classification of Satsuma mandarin as separated from Ponkan. The influence of blooming stages on cultivar clustering varied with individual citrus, was most obvious on hybrid citrus, and this should not be neglected when taking volatile profiles as a marker for revealing the genetic relationships between citrus cultivars.

## Supplementary Materials



## Figures and Tables

**Table 1:** Citrus cultivars included for the study of flower volatiles.

	Citrus types	Common name	Cultivars	Code
1	*C. reticulata* Blanco	Mandarin	Ponkan	PK
2	*C. unshiu* Marc.	Mandarin	Satsuma	ST
3	*C. sinensis*. (L.) Osbeck	Sweet orange	Liubencheng	LBC
4	*C. sinensis*. (L.) Osbeck	Sweet orange	Qingjia	QJ
5	*C. limon*. (L.) Burm.	Lemon	Eureka	ERK
6	*C. medica*. (L.)	Citron	Bergamot	BM
7	*C. grandis* (L.) Osbeck	Pomelo	Yuhuanyou	YHY
8	*C. grandis* (L.) Osbeck	Pomelo	Zaoxiangyou	ZXY
9	*C. changshanensis* Chen et. Fu (*C. aurantium × C. grandis*)	Citrus hybrid	Huyou	HY

**Table 2:** Identification of flower volatiles and quantification of their abundance by percentage in unopened, half opened and fully opened flowers from five citrus types.

RI a	Compound name	FC b	Ponkan			Satsuma			Qingjia			Liubencheng			Eureka			Bergamot			Yuhuanyou			Zaoxiangyou			Huyou		
								
			U^c^	H	F	U	H	F	U	H	F	U	H	F	U	H	F	U	H	F	U	H	F	U	H	F	U	H	F
	**Aldehydes**																												
809	Hexanal	A1	M	M	-d	-	-	-	-	-	-	-	-	M	M	M	M	-	-	M	M	M	M	M	M	M	3.44	M	M
858	2-Hexenal *	A2	M	-	M	2.84	1.84	M	2.36	1.68	1.19	1.51	1.20	1.86	1.33	1.45	M	M	M	M	M	M	1.68	M	M	1.24	5.95	1.52	M
977	Benzaldehyde	A3	T	T	M	T	M	T	T	T	T	-	-	M	T	T	T	-	-	-	-	T	T	-	-	-	M	T	-
1048	Benzene acetaldehyde *	A4	M	M	1.08	-	-	-	M	M	M	-	M	1.31	-	M	M	-	-	-	-	-	1.34	-	-	-	M	M	M
1109	Nonanal	A5	-	-	-	-	-	-	-	-	-	-	-	-	-	-	-	M	M	M	-	-	-	-	-	-	-	-	-
1154	Lilac aldehyde B *	A6	-	-	-	-	-	T	-	T	T	-	T	-	-	-	-	-	-	-	M	T	-	M	T	T	-	-	T
1202	Myrtenal	A7	-	-	-	M	M	-	-	-	-	T	-	-	-	-	-	-	-	-	-	T	-	T	-	T	-	T	-
1214	Decanal	A8	T	T	T	T	T	-	-	-	-	T	T	-	M	M	T	-	-	-	M	M	-	T	-	-	-	T	M
1311	Undecanal	A9	-	-	-	-	-	-	-	-	-	-	-	-	T	-	M	T	T	T	-	-	-	-	-	-	-	-	-
	**Monoterpene Hydrocarbons**																												
939	α-thujene	Mh1	M	M	M	M	M	M	M	M	M	M	M	M	T	T	M	M	M	M	-	T	2.18	T	T	T	M	M	M
945	α-pinene	Mh2	M	M	M	1.08	2.80	M	M	M	M	M	M	1.42	M	M	M	M	M	M	M	M	M	M	M	M	1.57	2.28	M
962	Camphene	Mh3	-	-	-	-	-	-	-	-	-	-	-	-	-	-	-	-	-	-	-	-	-	T	4.48	-	-	T	-
979	Sabinene	Mh4	-	-	-	-	-	-	11.15	6.07	6.11	-	-	-	-	-	-	-	-	-	-	-	-	-	-	-	-	-	-
991	β-pinene	Mh5	9.20	7.83	6.59	2.92	3.91	6.51	-	-	-	11.88	7.98	3.53	2.07	M	1.11	M	M	M	3.38	1.67	2.42	7.49	-	4.90	5.89	8.08	6.84
999	β-myrcene	Mh6	1.46	1.38	1.11	-	2.55	1.53	2.53	1.82	1.48	2.46	1.73	1.62	2.01	2.02	2.42	1.40	1.50	1.53	8.08	4.15	1.55	M	M	M	2.04	1.95	15.50
1018	α-terpinene	Mh7	M	M	M	M	M	M	M	-	M	1.62	-	M	-	-	-	M	M	M	T	T	M	-	-	-	M	M	M
1025	*p-*cymene	Mh8	M	M	M	8.56	6.53	-	-	-	-	-	-	M	-	-	-	-	-	-	-	-	1.01	-	-	-	2.91	-	-
1029	Limonene	Mh9	1.48	1.15	1.07	-	-	1.69	3.25	1.75	1.54	4.64	2.69	2.41	51.99	44.95	52.53	29.30	27.25	36.17	3.32	2.19	3.68	4.92	2.54	3.32	6.68	10.09	7.05
1040	(*Z*)-ocimene	Mh10	T	-	-				-	-	-	M	-	1.71	M	M	-	1.01	1.10	1.25	M	M	-	M	M	M	T	6.78	16.52
1044	(*E*)-ocimene	Mh11	3.03	2.29	2.16	6.37	6.32	2.27	1.62	1.18	1.39	2.95	1.54	8.40	6.14	5.35	6.35	2.74	2.56	2.96	6.41	3.23	1.97	9.14	5.45	8.33	4.90	-	-
1060	γ-terpinene	Mh12	1.90	1.44	1.63	13.79	8.91	M	M	M	M	M	M	M	2.60	1.97	3.17	3.68	3.71	4.29	M	T	11.06	T	T	T	18.07	22.83	T
1084	β-cymene *	Mh13	-	-	-	-	-	-	-	-	-	-	-	-	-	-	-	-	-	-	-	-	-	-	-	-	-	-	T
1090	Terpinolene	Mh14	-	-	-	-	-	-	M	M	M	M	M	M	M	M	-	M	M	M	-	-	-	-	-	-	1.07	1.59	M
1128	1,3,8-p-menthatriene *	Mh15	-	-	-	-	-	-	-	-	-	-	-	-	T	T	T	-	-	-	-	-	-	T	-	T	-	-	T
1131	2,4,6-octatriene,3,4-dimethyl *	Mh16	M	M	M	M	M	T	M	M	T	M	M	M	T	M	M	1.16	1.05	1.35	M	M	T	M	M	M	M	M	M
	**Oxygenated Monoterpenes**																												
1031	1,8-Cineol	Om1	-	-	-	6.05	3.15	-	-	-	-	-	-	-	-	-	-	-	-	-	-	-	-	-	-	-	-	-	-
1068	*cis*-β-terpineol *	Om2	1.99	1.65	1.08	M	M	M	3.60	1.81	M	2.93	1.77	-	M	T	T	M	M	M	T	T	M	T	T	T	M	M	T
1076	*cis*-linalol oxide *	Om3	-	-	-	-	-	-	-	T	-	-	-	M	-	-	-	-	-	-	M	M	T	M	M	M	-	-	-
1108	Linalool	Om4	46.76	50.43	47.74	22.67	17.41	42.76	44.74	42.91	46.98	29.15	36.11	24.95	7.95	6.88	3.94	1.45	1.72	1.59	54.41	56.16	21.59	36.44	53.55	48.43	18.83	10.10	30.11
1137	Limonene oxide, *cis*	Om5	-	-	-	-	-	-	-	-	-	-	-	-	-	-	-	-	-	-	-	T	1.15	-	-	-	-	-	T
1139	Limonene oxide, *trans*	Om6	-	-	-	-	-	-	-	-	-	-	-	-	2.58	-	3.04	1.63	2.19	2.78	-	-	-	-	-	-	-	-	-
1155	Citronellal	Om7	M	M	M	-	-	M	M	M	M	M	M	M	1.05	M	M	M	M	M	M	M	M	T	T	T	M	M	T
1176	Umbellulone *	Om8	-	T	-	-	-	-	-	-	-	-	-	-	-	-	-	-	-	-	-	-	-	-	-	-	-	-	-
1177	Terpinen-4-ol	Om9	M	M	M	M	M	M	M	M	M	M	M	M	M	-	M	T	T	T	M	M	M	M	M	M	M	M	T
1189	*p*-cymen-8-ol *	Om10	M	M	T	M	M	-	-	-	-	-	-	-	-	-	-	-	-	-	-	-	-	-	-	-	-	-	-
1192	α-terpineol	Om11	3.83	2.94	1.96	5.45	5.58	1.01	4.59	2.60	1.12	4.37	2.83	M	1.54	-	-	2.39	2.72	1.97	-	M	-	M	M	-	2.73	M	M
1205	*trans*-dihydrocarvone *	Om12	-	-	-	-	-	-	-	-	-	-	-	-	M	M	M	-	-	-	-	-	-	-	-	-	-	-	-
1208	Carvone *	Om13	-	-	-	-	-	-	-	-	-	-	-	-	-	-	-	T	T	T	-	-	-	-	-	-	-	-	T
1210	*trans*-piperitol *	Om14	-	-	-	-	-	-	-	-	-	-	-	-	-	-	-	-	-	-	-	-	-	-	-	-	-	-	T
1215	*p-*Menth-1-en-9-al *	Om15	T	T	M	M	-	-	-	-	-	-	-	-	-	M	M	-	-	-	-	-	-	M	M	M	-	-	-
1219	*cis*-carveol	Om16	-	-	-	1.00	M	M	-	-	-	T	-	-	M	M	M	M	M	1.15	T	T	-	T	T	T	-	T	T
1230	*cis*-geraniol	Om17	-	-	-	-	-	M	M	M	M	M	M	M	M	T	M	M	-	-	1.19	2.15	M	1.37	2.84	2.46	M	M	1.01
1233	β-citronellol	Om18	T	M	M	-	-	M	M	M	T	M	M	M	T	T	-	-	-	-	-	-	M	-	-	-	T	M	M
1236	Methyl thymyl ether	Om19	1.93	1.19	1.07	-	-	-	-	-	-	M	5.74	T	T	T	T	-	-	-	-	-	-	-	-	-	-	-	-
1237	Isogeraniol *	Om20	-	-	-	-	-	-	-	-	-	-	-	-	-	-	-	-	-	-	-	-	-	-	T	T	-	-	T
1242	β-citral	Om21	T	T	M	-	-	M	M	M	M	M	M	2.55	M	M	M	17.75	17.10	13.27	1.52	2.28	1.64	M	1.03	M	M	M	-
1258	*trans*-geraniol	Om22	M	1.12	2.47	M	-	-	T	-	-	T	M	6.80	-	-	-	M	M	M	M	1.82	6.52	M	1.20	1.24	M	2.45	M
1273	α-citral	Om23	M	T	T	-	-	M	M	M	M	M	11.17	3.30	1.24	M	M	25.81	24.50	20.07	1.83	3.07	2.40	M	1.19	M	M	1.38	M
1282	α-thujenal	Om24	-	-	-	M	1.33	M	-	-	-	-	-	-	-	-	-	-	-	-	-	-	-	-	-	-	-	-	-
1299	Carvacrol *	Om25	-	-	-	-	-	-	-	-	-	-	-	-	-	-	M	-	-	-	-	-	-	-	-	-	-	-	-
1733	E,E-farnesal *	Om26	-	-	-	-	-	M	-	-	-	-	-	-	T	-	M	T	M	T	-	-	M	M	M	M	-	M	M
	**Sesquiterpene Hydrocarbons**																												
1335	δ-elemene	Sh1	1.36	M	1.36	-	-	-	-	-	-	-	-	M	M	-	-	-	-	-	1.02	M	1.23	M	M	M	1.14	M	1.05
1336	α-cubebene *	Sh2	T	T	T	-	-	-	-	-	-	-	-	-	M	T	T	-	-	-	-	-	-	-	-	-	-	T	T
1371	Copaene *	Sh3	-	-	-	-	-	-	-	-	-	-	-	-	-	M	M	-	-	-	-	-	-	-	-	-	M	-	-
1392	β-elemene	Sh4	M	-	T	5.40	4.76	2.13	1.93	M	1.47	19.43	3.09	5.73	-	7.53	6.02	-	-	-	-	M	4.98	M	M	2.25	M	4.48	M
1398	Zingiberene *	Sh5	-	-	M	-	-	-	-	-	-	-	-	-	-	-	-	-	-	-	-	-	M	-	-	-	-	M	T
1407	Bergamotene *	Sh6	-	-	-	-	-	-	-	-	-	T	T	M	T	-	-	-	-	-	-	-	-	M	-	-	M	-	-
1408	*trans*-α-bergamotene	Sh7	M	T	-	-	-	M	M	M	T	-	-	-	-	T	T	-	-	-	-	-	-	-	-	-	-	-	-
1412	Caryophyllene	Sh8	M	M	M	2.11	2.61	-	M	M	M	1.02	M	1.34	3.50	3.93	3.14	1.83	1.99	1.79	1.13	0.70	2.18	T	-	T	2.31	2.98	2.00
1417	α-santalene *	Sh9	-	-	-	-	-	-	-	-	-	-	-	-	-	T	T	T	T	T	-	-	-	-	-	-	-	-	-
1422	β-cubebene	Sh10	T	T	T	-	M	M	-	-	-	-	-	0.56	T	-	T	T	T	T	0.08	0.06	0.25	-	1.64	-	M	M	M
1431	γ-elemene	Sh11	1.16	M	1.06	M	M	M	-	-	-	-	-	1.37	T	T	T	-	-	-	M	M	1.77	-	T	M	2.48	1.44	M
1435	α-bergamotene	Sh12	-	-	-	-	-	4.00	-	-	-	-	-	-	-	2.22	1.78	M	M	M	-	-	T	-	-	-	-	T	-
1445	α-caryophyllene	Sh13	-	-	-	-	-	-	M	-	-	-	-	-	-	M	M	-	-	-	-	-	-	-	-	-	-	-	-
1456	Bicyclosesquiphellandrene *	Sh14	M	M	M	-	-	-	M	M	-	M	M	M	-	-	-	T	T	T	-	T	-	M	M	T	-	-	T
1461	β-farnesene	Sh15	3.53	2.24	3.04	-	-	-	3.89	3.56	2.69	2.03	2.25	3.43	1.88	2.26	1.64	M	M	M	T	M	5.16	T	-	-	M	4.60	T
1464	α-gurjunene *	Sh16	-	-	-	-	-	-	-	-	-	-	-	-	-	-	-	-	-	-	-	-	-	-	-	-	-	T	-
1470	α-elemene *	Sh17	-	-	-	-	-	-	-	-	-	-	-	-	-	T	T	-	-	-	-	-	M	-	-	-	-	-	T
1474	Germacrene D	Sh18	M	M	T	M	M	T	-	-	-	-	-	1.36	M	M	M	M	M	M	M	M	1.17	-	M	M	2.24	1.52	1.14
1475	β-eudesmene *	Sh19	-	-	-	-	-	-	-	-	-	-	T	-	M	M	M	-	-	-	-	-	-	-	-	-	-	M	-
1478	α-selinene *	Sh20	-	-	-	T	M	-	-	-	-	-	-	M	-	-	M	-	-	-	-	-	M	-	-	-	2.04	-	-
1481	Allo-aromadendrene	Sh21	-	-	-	-	-	-	-	T	-	-	-	-	M	M	M	-	-	-	-	-	-	-	-	-	-	-	T
1489	Bicyclogermacrene	Sh22	-	-	-	M	M	T	-	-	-	T	-	-	-	-	-	-	-	-	T	M	-	M	M	M	-	-	-
1491	α-muurolene *	Sh23	-	M	M	-	-	-	-	-	-	-	-	-	-	-	-	-	-	-	-	-	M	-	-	T	-	-	-
1496	α-bulnesene *	Sh24	T	-	M	-	-	-	-	T	-	-	-	-	M	M	M	-	-	-	-	-	T	-	-	-	-	T	-
1499	(Z,E)-α-farnesene	Sh25	-	-	-	-	-	-	-	-	-	-	-	-	M	M	-	-	-	-	-	T	-	M	M	-	-	-	T
1502	γ-muurolene	Sh26	-	-	-	-	-	-	-	-	-	-	-	-	-	-	-	-	-	-	-	-	T	T	-	-	-	M	M
1503	α-farnesene *	Sh27	-	-	M	-	-	-	-	-	-	-	-	T	M	M	M	-	-	-	-	-	-	-	-	-	-	M	M
1504	β-bisabolene	Sh28	-	-	-	-	-	-	-	-	-	-	-	-	2.42	2.82	2.34	1.54	1.63	1.47	-	-	-	-	-	-	-	-	-
1506	δ-cadinene	Sh29	2.17	1.30	1.67	-	-	-	M	1.07	M	M	M	-	-	-	-	-	-	-	M	M	-	1.13	M	M	-	-	M
1507	β-sesquiphellandrene	Sh30	M	-	M	4.31	6.41	-	M	M	M	M	M	-	T	T	T	-	-	-	-	-	M	-	-	-	M	M	-
1510	Eudesma-3,7(11)-diene *	Sh31	-	-	-	-	-	-	-	-	-	-	-	M	-	-	-	-	-	-	-	-	-	-	-	-	-	T	T
1520	*cis*-α-bisabolene *	Sh32	-	-	-	-	-	-	-	-	-	-	-	-	T	-	T	M	M	M	-	-	T	-	-	-	-	-	-
	**Oxygenated Monoterpenes**																												
1530	Nerolidol	Os1	M	M	T	-	-	M	M	3.04	3.64	M	1.74	-	-	M	T	M	1.44	M	3.57	4.04	M	6.94	7.47	8.75	M	M	T
1532	Caryophyllene oxide	Os2	-	-	-	-	-	M	-	-	-	-	-	-	-	-	-	T	M	T	-	-	-	-	-	-	-	T	4.81
1658	β-eudesmol *	Os3	T	T	-	-	-	-	-	-	-	-	-	-	-	-	-	-	-	-	-	-	-	-	-	-	T	T	-
1696	Tetradecanal *	Os4	-	-	-	-	-	-	-	-	-	-	-	M	-	-	-	-	-	-	-	-	-	-	-	-	-	-	T
1734	Farnesol	Os5	-	M	M	M	1.07	M	M	1.54	M	1.41	1.11	-	M	M	M	M	M	M	1.32	1.73	M	2.38	2.28	2.00	M	M	-
1765	α-sinensal	Os6	M	M	M	-	-	-	-	-	-	M	M	-	-	-	-	-	-	-	-	-	-	-	-	-	-	-	-
	**Ketones**																												
1125	Chrysanthenone *	K1	-	-	-	M	M	-	M	-	-	-	-	-	-	-	-	-	-	-	-	-	-	-	-	-	-	-	-
1395	*cis*-jasmone	K2	M	M	M	M	1.44	M	M	M	M	M	M	M	T	M	M	-	-	-	-	-	M	-	-	-	M	M	-
	**Esters**																												
1306	Methyl geranate	E1	-	-	-	M	-	M	1.79	8.26	15.81	-	-	-	M	M	-	-	-	-	M	M	M	M	M	M	-	-	T
1347	Citronellyl acetate *	E2	-	-	-	-	-	-	-	-	-	-	-	-	-	T	T	-	-	-	-	-	-	-	-	-	-	T	T
1370	Nerol acetate	E3	-	-	-	-	-	T	T	T	T	T	T	-	-	-	-	T	T	T	M	M	M	M	M	M	-	-	-
1390	Geranyl acetate	E4	-	-	-	T	M	-	-	-	-	-	-	-	-	-	-	T	M	M	-	-	-	-	-	-	-	-	-
	**Miscellaneous**																												
1298	*p*-thymol	P1	4.96	4.21	4.03	-	M	-	-	-	-	-	-	-	M	1.05	-	-	-	-	-	-	T	-	-	-	-	-	-
1665	Methyl jasmonate *	DFG	-	-	-	-	-	-	-	-	-	-	-	T	-	-	-	-	-	-	T	T	-	-	-	-	-	-	-
1075	1-octanol	Ac1	-	-	-	-	-	-	-	-	-	-	-	-	T	T	T	T	-	T	-	-	-	-	-	-	T	T	T
1116	Phenylethyl alcohol	Ac2	T	M	M	M	M	M	-	M	M	-	T	M	-	-	-	-	-	-	M	M	M	M	M	M	-	M	-
897	Styrene *	Ah1	-	-	-	-	-	-	-	-	-	-	-	-	T	-	-	-	-	-	-	-	-	-	-	-	-	-	-
1091	α,*p*-dimethylstyrene	Ah2	M	M	M	2.17	1.33	-	-	-	-	-	-	-	-	-	-	-	-	-	M	M	M	-	-	-	-	-	-
1160	Naphthalene *	Ah3	-	-	-	-	-	-	-	-	-	-	-	-	-	-	M	-	-	-	-	-	-	-	-	-	-	-	-
1140	Benzyl nitrile	N1	M	2.14	4.61	1.99	3.43	1.20	1.02	1.38	1.25	M	M	2.49	-	-	-	-	-	-	M	M	-	M	M	M	M	-	M
1292	Indole	N2	2.28	3.96	4.99	3.69	5.00	4.90	5.27	10.41	4.86	4.45	6.91	8.43	M	1.01	M	T	M	M	4.79	5.51	8.84	8.16	7.18	7.88	3.72	4.52	2.83
1341	Methyl anthranilate	N3	-	M	M	1.12	4.87	17.91	-	-	-	-	-	1.77	M	2.47	T	-	-	-	-	4.20	2.79	8.21	-	-	-	-	2.17
1535	Pentadecane, 3-methyl-*	Ak1	-	-	-	-	-	2.56	-	-	-	M	T	1.14	-	T	T	-	-	-	-	-	-	-	-	-	-	-	-
1680	Hexadecane, 2-methyl-*	Ak2	-	-	-	-	-	T	M	M	M	T	M	-	-	-	-	-	-	-	-	-	-	-	-	-	-	T	-
1689	8-heptadecene	Ak3	2.23	2.62	1.70	M	M	1.26	1.54	2.43	1.74	M	2.76	T	M	1.65	M	M	M	M	-	-	1.03	-	-	-	1.59	M	-
1809	Octadecane, 2-methyl *	Ak4	T	T	T	-	-	-	M	M	T	M	T	T	-	-	-	-	-	-	-	M	-	M	M	M	-	-	T

Data are arranged according to chemical groups, represented in mean percentage (peak area percentage) of individual flower constituents from triplicate experiments; letter indicated by; T: Trace (<0.1%), M: moderate (between 0.1% and 1%).

* Tentatively identified new compounds;

a Retention indexes analyzed on HP-5MS column;

b Family code. A: Aldehydes; Mh: Monoterpene hydrocarbons; Om: Oxygenated monoterpenes; Sh: Sesquiterpene hydrocarbons; Os: Oxygenated Sesquiterpenes; K: Ketones; E: Esters; P: Phenol; DFG: Diverse functional group; Ac: Alcohol; Ah: Aromatic Hydrocarbons; N: Nitrogen Derivatives; Ak: Alkanes;

c Flower stages are represented as, U; Unopened flower; H; Half opened flower, F; Fully opened flower;

d Undetectable.

**Table 3:** Major volatiles compounds, in percentages and rank, from unopened flowers from nine citrus cultivars.

Compounds	FC a	PK b	ST	LBC	QJ	ERK	BM	YHY	ZXY	HY	Mean rank
Linalool	Om4	46.76 ^1st^	22.67 ^1st^	44.74 ^1st^	29.15 ^1st^	7.95 ^2nd^	1.45 ^10th^	54.41 ^1st^	36.44 ^1st^	18.83 ^1st^	29.16 ^1st^
Limonene	Mh9	1.48 ^13th^	- c	3.25 ^7th^	4.64 ^4th^	51.99 ^1st^	29.30 ^1st^	3.32 ^7th^	4.92 ^7th^	6.68 ^3rd^	11.73 ^2nd^
β-pinene	Mh5	9.20 ^2nd^	2.92 ^10th^	-	11.88 ^3rd^	2.07 ^8th^	0.54 ^18th^	3.38 ^6th^	7.49 ^5th^	5.89 ^5th^	4.82 ^3rd^
(*E*)-ocimene	Mh11	3.03 ^6th^	6.37 ^4th^	1.62 ^12th^	2.95 ^7th^	6.14 ^3rd^	2.74 ^5th^	6.14 ^3rd^	9.14 ^3rd^	4.90 ^6th^	4.81 ^4th^
γ-terpinene	Mh12	1.90 ^12th^	13.79 ^2nd^	0.53 ^22nd^	0.53 ^21st^	2.60 ^5th^	3.68 ^4th^	0.11 ^34th^	0.09 ^39th^	18.07 ^2nd^	4.59 ^5th^
Indole	N2	2.28 ^7th^	3.69 ^9th^	5.27 ^3rd^	4.45 ^5th^	0.63 ^19th^	0.08 ^41st^	4.79 ^4th^	8.16 ^4th^	3.72 ^7th^	3.67 ^6th^
α-citral	Om23	0.12 ^41st^	-	0.68 ^19th^	0.59 ^18th^	1.24 ^14th^	25.81 ^2nd^	1.83 ^8th^	0.92 ^14th^	0.45 ^28th^	3.52 ^7th^
β-elemene	Sh4	0.39 ^27th^	5.40 ^7th^	1.93 ^10th^	19.43 ^2nd^	-	-	-	0.26 ^30th^	0.42 ^29th^	3.09 ^8th^
α-terpineol	Om11	3.83 ^4th^	5.45 ^6th^	4.59 ^4th^	4.37 ^6th^	1.54 ^12th^	2.39 ^6th^	-	0.42 ^23th^	2.73 ^10th^	2.81 ^9th^
β-citral	Om21	0.04 ^53th^	-	0.48 ^23rd^	0.43 ^23rd^	0.92 ^17th^	17.75 ^3rd^	1.52 ^9th^	0.93 ^13th^	0.30 ^33rd^	2.49 ^10th^
β-myrcene	Mh6	1.46 ^14th^	-	2.53 ^8th^	2.46 ^9th^	2.01 ^9th^	1.40 ^11th^	8.08 ^2nd^	0.98 ^11th^	2.04 ^14th^	2.33 ^11th^
2-hexenal	A2	0.62 ^20th^	2.84 ^11th^	2.36 ^9th^	1.51 ^12th^	1.33 ^13th^	0.64 ^17th^	0.93 ^14th^	0.88 ^15th^	5.95 ^4th^	1.90 ^12th^
Caryophyllene	Sh8	0.48 ^23rd^	2.11 ^13th^	0.65 ^20th^	1.02 ^14th^	3.50 ^4th^	1.83 ^7th^	1.13 ^12th^	0.07 ^43rd^	2.31 ^12th^	1.46 ^13th^
*p*-cymene	Mh8	0.56 ^21st^	8.56 ^3rd^	-	-		-	-	-	2.91 ^9th^	1.34 ^14th^
β-farnesene	Sh15	0.13 ^40th^	-	0.29 ^39th^	0.26 ^45th^	-	0.27 ^31th^	3.57 ^5th^	0.06 ^45th^	0.10 ^42th^	1.31 ^15th^

a Family code;

b The full names corresponding to the abbreviations are as indicated in Table 1;

c Undetectable.

**Table 4:** Major volatiles compounds, in percentages and rank, from half opened flowers from nine citrus cultivars.

Compounds	FC a	PK b	ST	LBC	QJ	ERK	BM	YHY	ZXY	HY	Mean rank
Linalool	Om4	50.43 ^1st^	17.41 ^1st^	42.91 ^1st^	36.11 ^1st^	6.88 ^3rd^	1.72 ^9th^	56.16 ^1st^	53.55 ^1st^	10.10 ^2nd^	30.58 ^1st^
Limonene	Mh9	1.151 ^5th^	- c	1.75 ^11th^	2.69 ^9th^	44.95 ^1st^	27.25 ^1st^	2.19 ^9th^	2.54 ^7th^	10.09 ^3rd^	10.29 ^2nd^
Indole	N2	3.96 ^4th^	5.00 ^6th^	10.41 ^2nd^	6.91 ^4th^	1.01 ^15th^	0.16 ^32nd^	5.51 ^2nd^	7.18 ^3rd^	4.52 ^7th^	4.96 ^3rd^
α-citral	Om23	0.045 ^1st^	-	0.66 ^19th^	11.17 ^2nd^	0.911 ^8th^	24.50 ^2nd^	3.07 ^7th^	1.19 ^11th^	1.38 ^17th^	4.77 ^4th^
γ-terpinene	Mh12	1.44 ^11th^	8.91 ^2nd^	0.30 ^27th^	0.32 ^29th^	1.97 ^11th^	3.71 ^4th^	0.05 ^43rd^	0.05 ^42nd^	22.83 ^1st^	4.40 ^5th^
β-pinene	Mh5	7.83 ^2nd^	3.91 ^9th^	-	7.98 ^3rd^	0.81 ^19th^	0.68 ^18th^	1.67 ^13th^	-	8.08 ^4th^	3.44 ^6th^
β-citral	Om21	0.094 ^3rd^	-	0.33 ^26th^	0.26 ^31th^	0.65 ^22nd^	17.10 ^3rd^	2.28 ^8th^	1.03 ^12th^	0.841 ^9th^	2.51 ^7th^
β-elemene	Sh4	-	4.76 ^8th^	0.12 ^38th^	3.09 ^6th^	7.53 ^2nd^	-	0.14 ^29th^	0.19 ^29th^	4.48 ^8th^	2.26 ^8th^
Methyl anthranilate	N3	0.22 ^34th^	4.87 ^7th^	8.26 ^3rd^	-	2.47 ^7th^	-	4.20 ^3rd^	-	-	2.22 ^9th^
Nerolidol	Os4	0.24 ^32nd^	-	3.04 ^6th^	1.74 ^12th^	0.15 ^38th^	1.44 ^12th^	4.04 ^5th^	7.47 ^2nd^	0.40 ^30th^	2.06 ^10th^
β-Myrcene	Mh6	1.38 ^12th^	2.55 ^16th^	1.82 ^9th^	1.73 ^13th^	2.02 ^10th^	1.50 ^11th^	4.15 ^4th^	0.89 ^13th^	1.95 ^12th^	2.00 ^11th^
α-terpineol	Om11	2.94 ^5th^	5.58 ^5th^	2.60 ^7th^	2.83 ^7th^	-	2.72 ^5th^	0.42 ^17th^	0.19 ^28th^	0.50 ^27th^	1.98 ^12th^
(*E*)-ocimene	Mh11	2.29 ^7th^	2.81 ^13th^	1.18 ^15th^	1.54 ^14th^	-	2.56 ^6th^	-	5.45 ^4th^	-	1.76 ^13th^
β-farnesene	Sh15	2.24 ^8th^	-	3.56 ^5th^	2.25 ^10th^	2.26 ^8th^	0.13 ^37th^	0.10 ^39th^	-	4.60 ^6th^	1.68 ^14th^
Caryophyllene	Sh8	0.33 ^31th^	2.61 ^15th^	0.56 ^21th^	0.93 ^17th^	3.93 ^5th^	1.99 ^8th^	0.70 ^14th^	-	2.98 ^9th^	1.56 ^15th^

a Family code;

b The full names corresponding to the abbreviations are as indicated in Table 1;

c Undetectable.

**Table 5:** Major volatiles compounds, in percentages and rank, from fully opened flowers from nine citrus cultivars.

Compounds	FC a	PK b	ST	LBC	QJ	ERK	BM	YHY	ZXY	HY	Mean rank
Linalool	Om4	47.74 ^1st^	42.76 ^1st^	46.98 ^1st^	24.95 ^1st^	3.94 ^4th^	1.59 ^9th^	21.59 ^1st^	48.43 ^1st^	30.11 ^1st^	29.79 ^1st^
Limonene	Mh9	1.071 ^8th^	1.69 ^8th^	1.54 ^8th^	2.41 ^11th^	52.53 ^1st^	36.17 ^1st^	3.68 ^7th^	3.32 ^6th^	7.05 ^4th^	12.16 ^2nd^
Indole	N2	4.99 ^3rd^	4.90 ^4th^	4.86 ^4th^	8.43 ^2nd^	0.73 ^16th^	0.14 ^33th^	8.84 ^3rd^	7.88 ^4th^	2.83 ^7th^	4.84 ^3rd^
Methyl anthranilate	N3	0.342 ^9th^	17.91 ^2nd^	15.81 ^2nd^	1.77 ^13th^	0.04 ^53th^	- c	2.79 ^8th^	-	2.17 ^8th^	4.54 ^4th^
β-pinene	Mh5	6.59 ^2nd^	6.51 ^3rd^	-	3.53 ^6th^	1.11 ^12th^	0.60 ^17th^	2.42 ^9th^	4.90 ^5th^	6.84 ^5th^	3.61 ^5th^
α-citral	Om23	0.074 ^8th^	0.43 ^23th^	0.78 ^18th^	3.30 ^8th^	0.50 ^24th^	20.07 ^2nd^	2.40 ^10th^	0.76 ^14th^	0.84 ^14th^	3.24 ^6th^
β-myrcene	Mh6	1.11 ^14th^	1.53 ^9th^	1.48 ^9th^	1.62 ^15th^	2.42 ^8th^	1.53 ^10th^	1.55 ^17th^	0.99 ^12th^	15.50 ^3rd^	3.08 ^7th^
(*E*)-ocimene	Mh11	2.16 ^8th^	0.93 ^15th^	1.39 ^11th^	8.40 ^3rd^	-	2.96 ^15th^	-	8.33 ^3rd^	-	2.69 ^8th^
β-elemene	Sh5	0.02 ^58th^	2.13 ^7th^	1.47 ^10th^	5.73 ^5th^	6.02 ^3rd^	-	4.98 ^6th^	2.25 ^8th^	0.34 ^23rd^	2.55 ^9th^
γ-terpinene	Mh12	1.63 ^12th^	0.18 ^34th^	0.16 ^29th^	0.12 ^42th^	3.17 ^5th^	4.29 ^4th^	11.06 ^2nd^	0.04 ^41th^	0.08 ^35th^	2.30 ^10th^
(*Z*)-ocimene	Mh10	-	-	-	1.71 ^14th^	-	1.25 ^13th^	-	0.22 ^26th^	16.52 ^2nd^	2.19 ^11th^
β-citral	Om21	0.15 ^39th^	0.29 ^28th^	0.28 ^25th^	2.55 ^9th^	0.52 ^23th^	13.27 ^3rd^	1.64 ^16th^	0.72 ^15th^	-	2.16 ^12th^
β-farnesene	Sh15	3.04 ^6th^	-	2.69 ^6th^	3.43 ^7th^	1.64 ^11th^	0.11 ^36th^	5.16 ^5th^	-	0.09 ^34th^	1.80 ^13th^
Nerolidol	Os1	0.06 ^49th^	0.25 ^29th^	3.64 ^5th^	-	0.064 ^5th^	0.51 ^19th^	0.92 ^24th^	8.75 ^2nd^	0.02 ^59th^	1.58 ^14th^
Caryophyllene	Sh8	0.42 ^25th^	-	0.43 ^21st^	1.34 ^20th^	3.14 ^6th^	1.79 ^8th^	2.18 ^11th^	0.05 ^40th^	2.00 ^9th^	1.26 ^15th^

a Family code;

b The full names corresponding to the abbreviations are as indicated in Table 1;

c Undetectable.

## References

[1] Colquhoun T.A., Clark D.G. (2011). Unraveling the regulation of floral fragrance biosynthesis. Plant Signal Behav.

[2] Schiestl F.P. (2010). The evolution of floral scent and insect chemical communication. Ecol. Lett.

[3] Raguso R.A. (2008). Wake up and smell the roses: The ecology and evolution of floral scent. Annu. Rev. Ecol. Evol. Syst.

[4] Pichersky E., Gershenzon J. (2002). The formation and function of plant volatiles: Perfumes for pollinator attraction and defense. Curr. Opin. Plant Biol.

[5] Unsicker S.B., Kunert G., Gershenzon J. (2009). Protective perfumes: The role of vegetative volatiles in plant defense against herbivores. Curr. Opin. Plant Biol.

[6] Dudareva N., Andersson S., Orlova I., Gatto N., Reichelt M., Rhodes D., Boland W., Gershenzon J. (2005). The nonmevalonate pathway supports both monoterpene and sesquiterpene formation in snapdragon flowers. Proc. Natl. Acad. Sci. USA.

[7] Chrubasik C., Roufogalis B.D., Muller Ladner U., Chrubasik S. (2008). A systematic review on the *Rosa canina* effect and efficacy profiles. Phytother. Res.

[8] Pichersky E., Noel J.P., Dudareva N. (2006). Biosynthesis of plant volatiles: Nature’s diversity and ingenuity. Sci. Signal.

[9] Knudsen J.T., Eriksson R., Gershenzon J., Ståhl B. (2006). Diversity and distribution of floral scent. Bot. Rev.

[10] Knudsen J.T., Tollsten L., Bergström L.G. (1993). Floral scents-a checklist of volatile compounds isolated by head-space techniques. Phytochemistry.

[11] Buckinham J. Dictionary of natural products.

[12] Liang P.H., Ko T.P., Wang A.H.J. (2002). Structure, mechanism and function of prenyltransferases. Eur. J. Biochem.

[13] Okada K., Kasahara H., Yamaguchi S., Kawaide H., Kamiya Y., Nojiri H., Yamane H. (2008). Genetic evidence for the role of isopentenyl diphosphate isomerases in the mevalonate pathway and plant development in Arabidopsis. Plant Cell Physiol.

[14] Allemann R.K. (2008). Chemical wizardry? The generation of diversity interpenoid biosynthesis. Pure Appl. Chem.

[15] Degenhardt J., Köllner T.G., Gershenzon J. (2009). Monoterpene and sesquiterpene synthases and the origin of terpene skeletal diversity in plants. Phytochemistry.

[16] Schmidt M.R., Wei W. (2006). Loss of agro–biodiversity, uncertainty, and perceived control: A comparative risk perception study in Austria and China. Risk Anal.

[17] Swingle W. The botany of citrus and its wild relatives of the orange subfamily.

[18] Liu C., Jiang D., Cheng Y., Deng X., Chen F., Fang L., Ma Z., Xu J. (2013). Chemotaxonomic study of *Citrus, Poncirus* and *Fortunella* genotypes based on peel oil volatile compounds-deciphering the genetic origin of Mangshanyegan (*Citrus nobilis* Lauriro). PLoS One.

[19] Tanaka T. (1954). Species problem in citrus: A critical study of wild and cultivated units of citrus based upon field studies in their native homes (Revisio Aurantiacearum IX). Jpn. Soc. Promot. Sci. Tokyo Jpn..

[20] Moore G.A. (2001). Oranges and lemons: Clues to the taxonomy of Citrus from molecular markers. Trends Genet.

[21] Azam M., Qian J., Bo Z., Changjie X., Kunsong C. (2013). Citrus leaf volatiles as affected by developmental stage and genotype. Int. J. Mol. Sci.

[22] Boussaada O., Chemli R. (2007). Seasonal variation of essential oil composition of *Citrus aurantium* L. var. amara. J. Essent. Oil Bear Plants.

[23] González-Mas M.C., Rambla J.L., Alamar M.C., Gutiérrez A., Granell A. (2011). Comparative analysis of the volatile fraction of fruit juice from different *Citrus* species. PLoS One.

[24] Jabalpurwala F.A., Smoot J.M., Rouseff R.L. (2009). A comparison of citrus blossom volatiles. Phytochemistry.

[25] Hou Y., Liang W., Zhang L., Cheng S., He F., Wu Z. (2011). Fresh water algae chemotaxonomy by high-performance liquid chromatographic (HPLC) analysis. Front. Environ. Sci. Engin. China.

[26] Li R., Luo G., Meyers P.A., Gu Y., Wang H., Xie S. (2012). Leaf wax n-alkane chemotaxonomy of bamboo from a tropical rain forest in Southwest China. Plant Syst. Evol.

[27] Ozel M., Gogus F., Hamilton J., Lewis A. (2004). The essential oil of *Pista ciavera* L. at various temperatures of direct thermal desorption using comprehensive gas chromatography coupled with time-of-flight mass spectrometry. Chromatographia.

[28] Pellati F., Benvenuti S., Melegari M. (2005). Chromatographic performance of a new polar poly(ethylene glycol) bonded phase for the phytochemical analysis of *Hypericum perforatum* L. J. Chromatogr.

[29] Kim N.-S., Lee D.-S. (2002). Comparison of different extraction methods for the analysis of fragrances from *Lavandula* species by gas chromatography-mass spectrometry. J. Chromatogr.

[30] Li Z.-G., Lee M.-R., Shen D.-L. (2006). Analysis of volatile compounds emitted from fresh *Syringa oblata* flowers in different florescence by headspace solid-phase microextraction–gas chromatography-mass spectrometry. Anal. Chim. Acta.

[31] Bartak P., Bednář P., Čáp L., Ondrakova L., Stránský Z. (2003). SPMEA valuable tool for investigation of flower scent. J. Sep. Sci.

[32] Flamini G., Cioni P.L., Morelli I. (2003). Use of solid-phase micro-extraction as a sampling technique in the determination of volatiles emitted by flowers, isolated flower parts and pollen. J. Chromatogr. A.

[33] Raguso R.A. (2004). Why are some floral nectars scented?. Ecology.

[34] Miyazaki T., Plotto A., Goodner K., Gmitter F.G. (2011). Distribution of aroma volatile compounds in tangerine hybrids and proposed inheritance. J. Sci. Food Agric.

[35] Alissandrakis E., Daferera D., Tarantilis P.A., Polissiou M., Harizanis P.C. (2003). Ultrasound-assisted extraction of volatile compounds from citrus flowers and citrus honey. Food Chem.

[36] Choi H.-S. (2003). Characterization of *Citrus unshiu* (*C. unshiu* Marcov. forma Miyagawa-wase) blossom aroma by solid-phase microextraction in conjunction with an electronic nose. J. Agric. Food Chem.

[37] Flamini G., Tebano M., Cioni P.L. (2007). Volatiles emission patterns of different plant organs and pollen of Citrus limon. Anal. Chim. Acta.

[38] Darjazi B.B. (2011). Comparison of volatile components of flower, leaf, peel and juice of ‘Page’ mandarin [(Citrus *reticulata* var ‘Dancy’ × Citrus paradisi var ‘Duncan’) × Citrus clementina]. Afr. J. Biotech.

[39] Darjazi B.B. (2012). A comparison of volatile components of flower, leaf and peel of *Citrus reticulata* Blanco (*Citrus nobilis* Lour var. deliciosa Swingle). J. Med. Plants Res.

[40] Xu C., Bao L., Zhang B., Bei Z., Ye X., Zhang S., Chen K. (2006). Parentage analysis of Huyou (*Citrus changshanensis*) based on internal transcribed spacer sequences. Plant Breed.

[41] Hosni K., Hassen I., Sebei H., Casabianca H. (2013). Secondary metabolites from *Chrysanthemum coronarium* (Garland) flower heads: Chemical composition and biological activities. Ind. Crop Prod.

[42] Flamini G., Cioni P.L. (2010). Odour gradients and patterns in volatile emission of different plant parts and developing fruits of grapefruit (*Citrus paradisi* L.). Food Chem.

[43] Lin S.Y., Roan S.F., Lee C.L., Chen I.Z. (2010). Volatile organic components of fresh leaves as indicators of indigenous and cultivated citrus Species in Taiwan. Biosci. Biotechnol. Biochem.

[44] Shalit M., Guterman I., Volpin H., Bar E., Tamari T., Menda N., Adam Z., Zamir D., Vainstein A., Weiss D. (2003). Volatile ester formation in roses. Identification of an acetyl-coenzyme A. Geraniol/Citronellol acetyl transferase in developing rose petals. Plant Physiol.

[45] Tanaka T. (1969). Misunderstanding with regards citrus classification and nomenclature. Bull. Univ. Osaka Prefect Ser. B.

[46] Coletta Filho H., Machado M., Targon M., Moreira M., Pompeu J. (1998). Analysis of the genetic diversity among mandarins (*Citrus* spp.) using RAPD markers. Euphytica.

[47] Federici C., Fang D., Scora R., Roose M. (1998). Phylogenetic relationships within the genus Citrus (*Rutaceae*) and related genera as revealed by RFLP and RAPD analysis. Theor. Appl. Genet.

[48] Kitajima A., Yamasaki A., Habu T., Preedasuttijit B., Hasegawa K. (2007). Chromosome identification and karyotyping of Satsuma mandarin by genomic *in situ* hybridization. J. Am. Soc. Hort. Sci.

[49] Luro F.L., Costantino G., Terol J., Argout X., Allario T., Wincker P., Talon M., Ollitrault P., Morillon R. (2008). Transferability of the EST-SSRs developed on Nules clementine (*Citrus clementina* Hort ex Tan) to other Citrus species and their effectiveness for genetic mapping. BMC Genomics.

[50] Zhang B., Xi W., Wei W., Shen J., Ferguson I., Chen K. (2011). Changes in aroma-related volatiles and gene expression during low temperature storage and subsequent shelf-life of peach fruit. Postharvest. Biol. Technol.

