# Supplementary material for: Comparative Analysis of Flower Volatiles from Nine Citrus at Three Blooming Stages
Source: Int J Mol Sci. 2013 Nov 13;14(11):22346–67. doi: 10.3390/ijms141122346 (PMC3856067; doi:10.3390/ijms141122346)
Supplement: Supplementary file 1 [file ijms-14-22346-s001.pdf]

Supplementary Materials

Figure S1. Chromatogram of E-Nose and gaschromatogarphy-mass spectrometry (GC-MS) from nine cultivars during flower development.

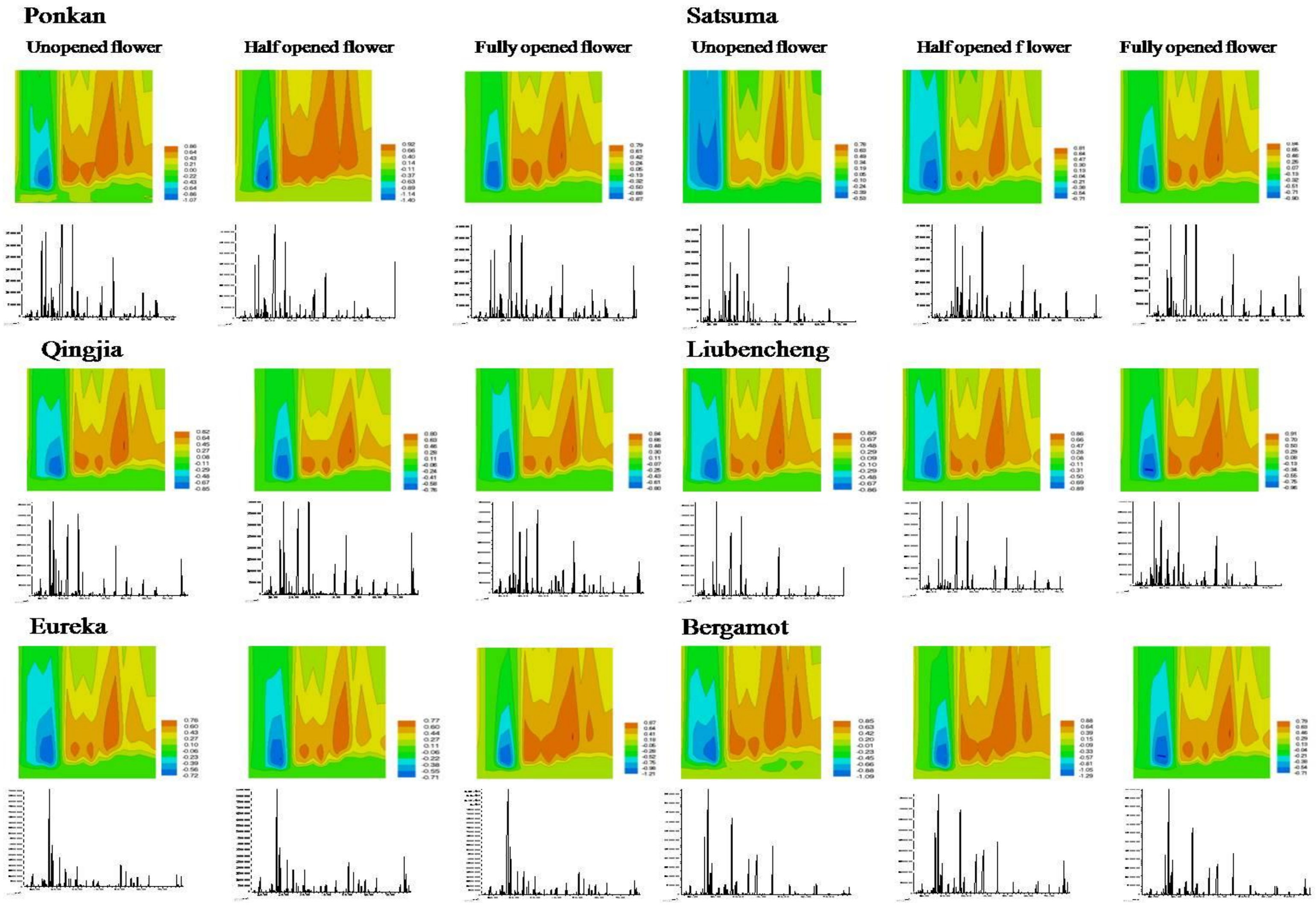

Figure S1. *Cont.*

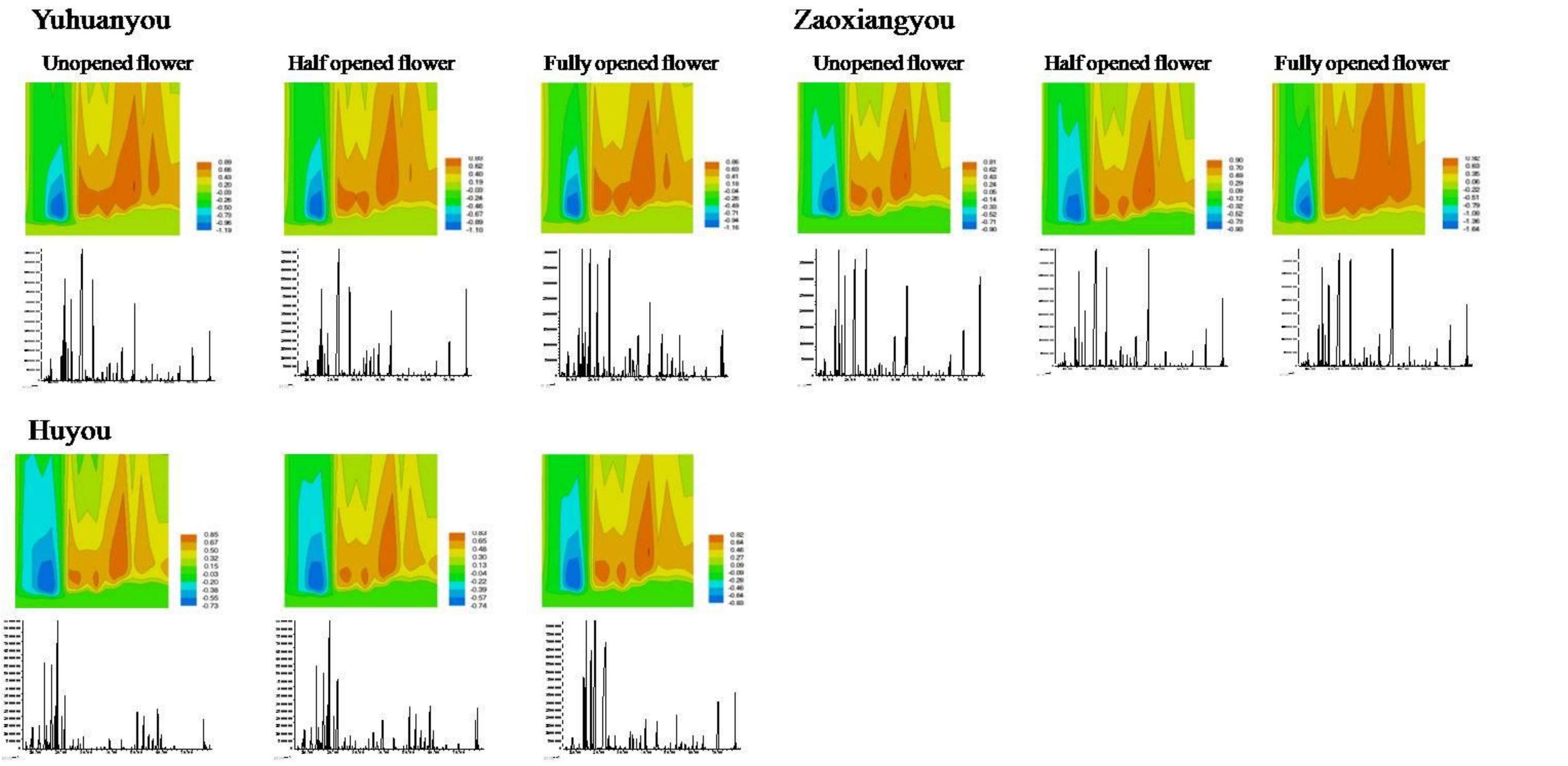

© 2013 by the authors; licensee MDPI, Basel, Switzerland. This article is an open access article distributed under the terms and conditions of the Creative Commons Attribution license (http://creativecommons.org/licenses/by/3.0/).
